# Supplementary material for: EMS Derived Wheat Mutant BIG8-1 (Triticum aestivum L.)—A New Drought Tolerant Mutant Wheat Line
Source: Int J Mol Sci. 2021 May 18;22(10):5314. doi: 10.3390/ijms22105314 (PMC8158095; doi:10.3390/ijms22105314)
Supplement: Supplementary file 1 [file ijms-22-05314-s001.zip › ijms-1170725-supplementary/Supplementary/Supplementary Fig S3 Venn diagramme.pptx]

## Slide 1
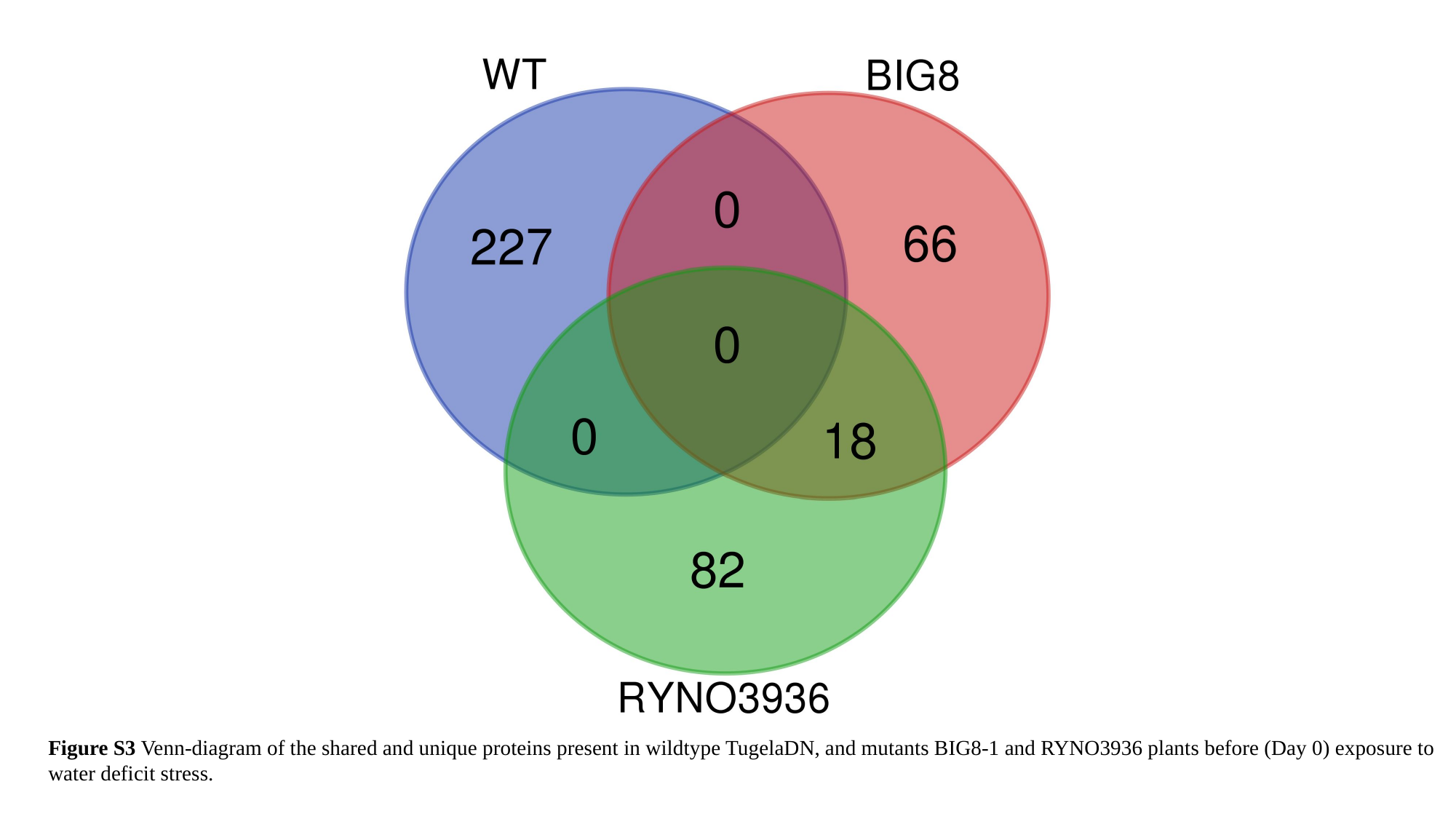

Figure S3 Venn-diagram of the shared and unique proteins present in wildtype TugelaDN, and mutants BIG8-1 and RYNO3936 plants before (Day 0) exposure to water deficit stress.
