# Supplementary material for: EMS Derived Wheat Mutant BIG8-1 (Triticum aestivum L.)—A New Drought Tolerant Mutant Wheat Line
Source: Int J Mol Sci. 2021 May 18;22(10):5314. doi: 10.3390/ijms22105314 (PMC8158095; doi:10.3390/ijms22105314)
Supplement: Supplementary file 1 [file ijms-22-05314-s001.zip › ijms-1170725-supplementary/Supplementary/Supplementary Figures S1_2 Cluster and FC .pptx]

## Slide 1
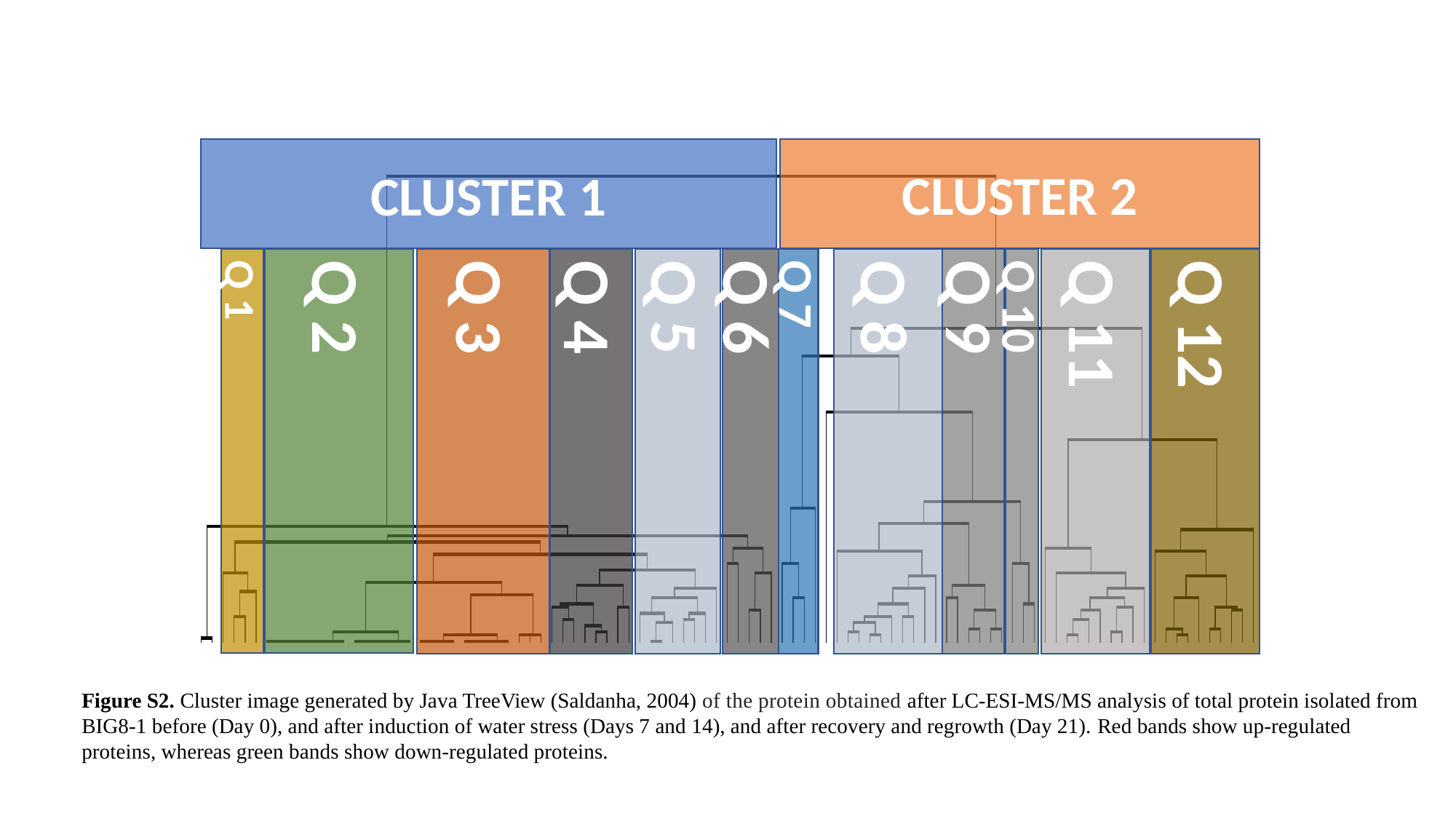

CLUSTER 1
CLUSTER 2
Q 6
Q 7
Q 8
Q 9
Q 10
Q 11
Q 12
Q 5
Q 1
Q 2
Q 3
Q 4
Figure S2. Cluster image generated by Java TreeView (Saldanha, 2004) of the protein obtained after LC-ESI-MS/MS analysis of total protein isolated from BIG8-1 before (Day 0), and after induction of water stress (Days 7 and 14), and after recovery and regrowth (Day 21). Red bands show up-regulated proteins, whereas green bands show down-regulated proteins.

## Slide 2
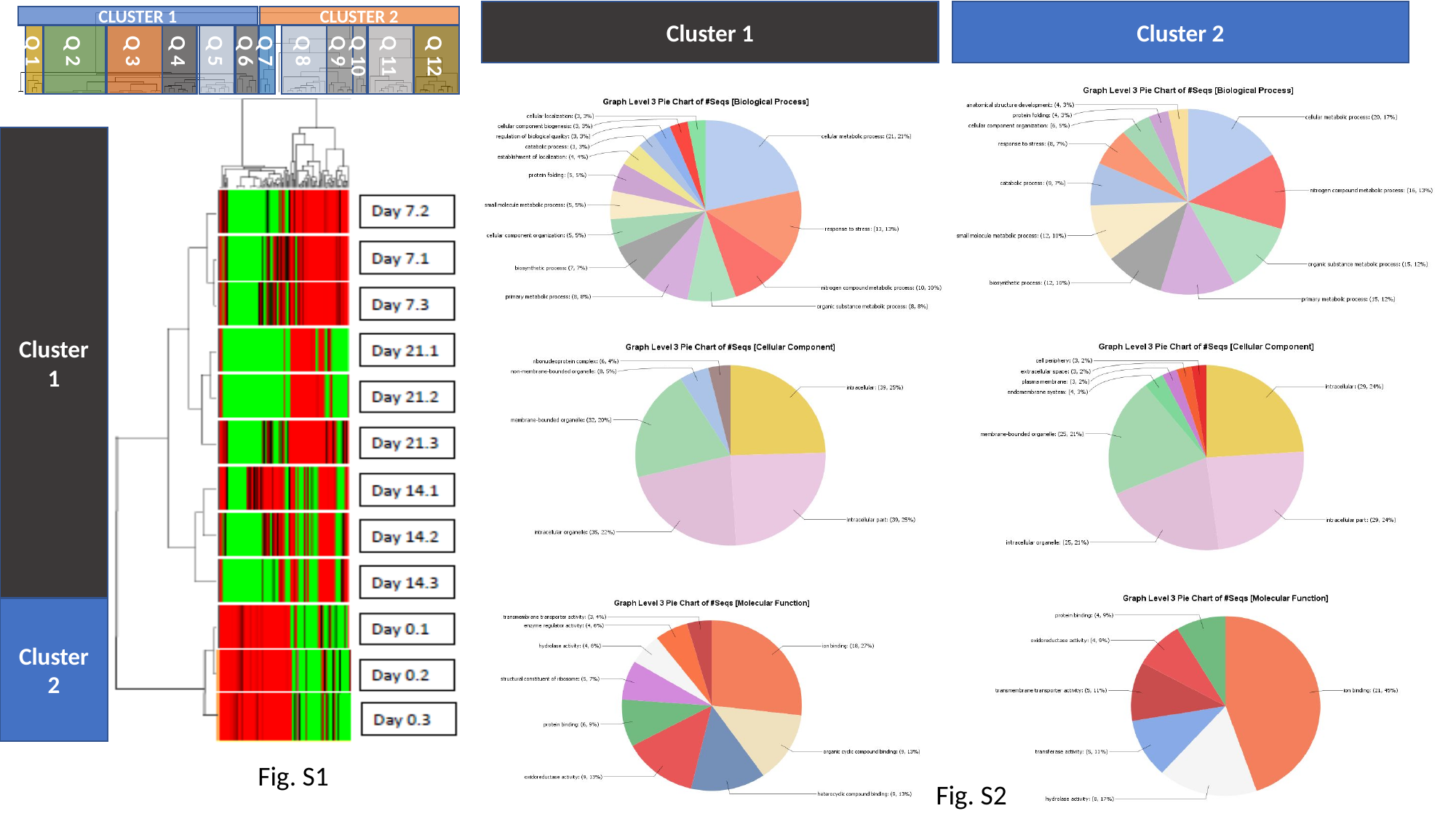

Cluster 1
Cluster 2
CLUSTER 1
CLUSTER 2
Q 6
Q 7
Q 8
Q 9
Q 10
Q 11
Q 12
Q 5
Q 1
Q 2
Q 3
Q 4
Cluster 1
Cluster 2
Fig. S1
Fig. S2

## Slide 3
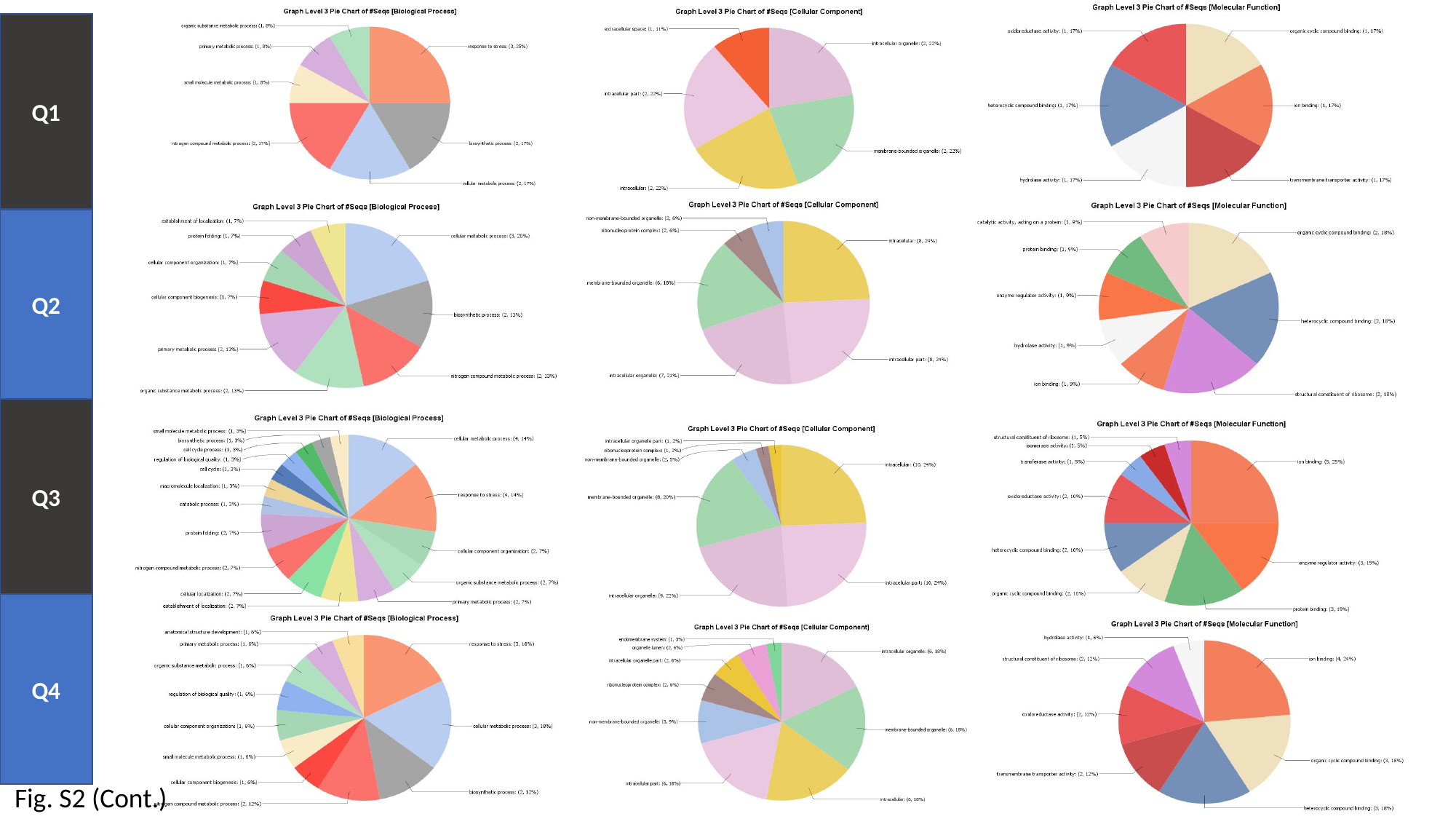

Q1
Q2
Q3
Q4
Fig. S2 (Cont.)

## Slide 4
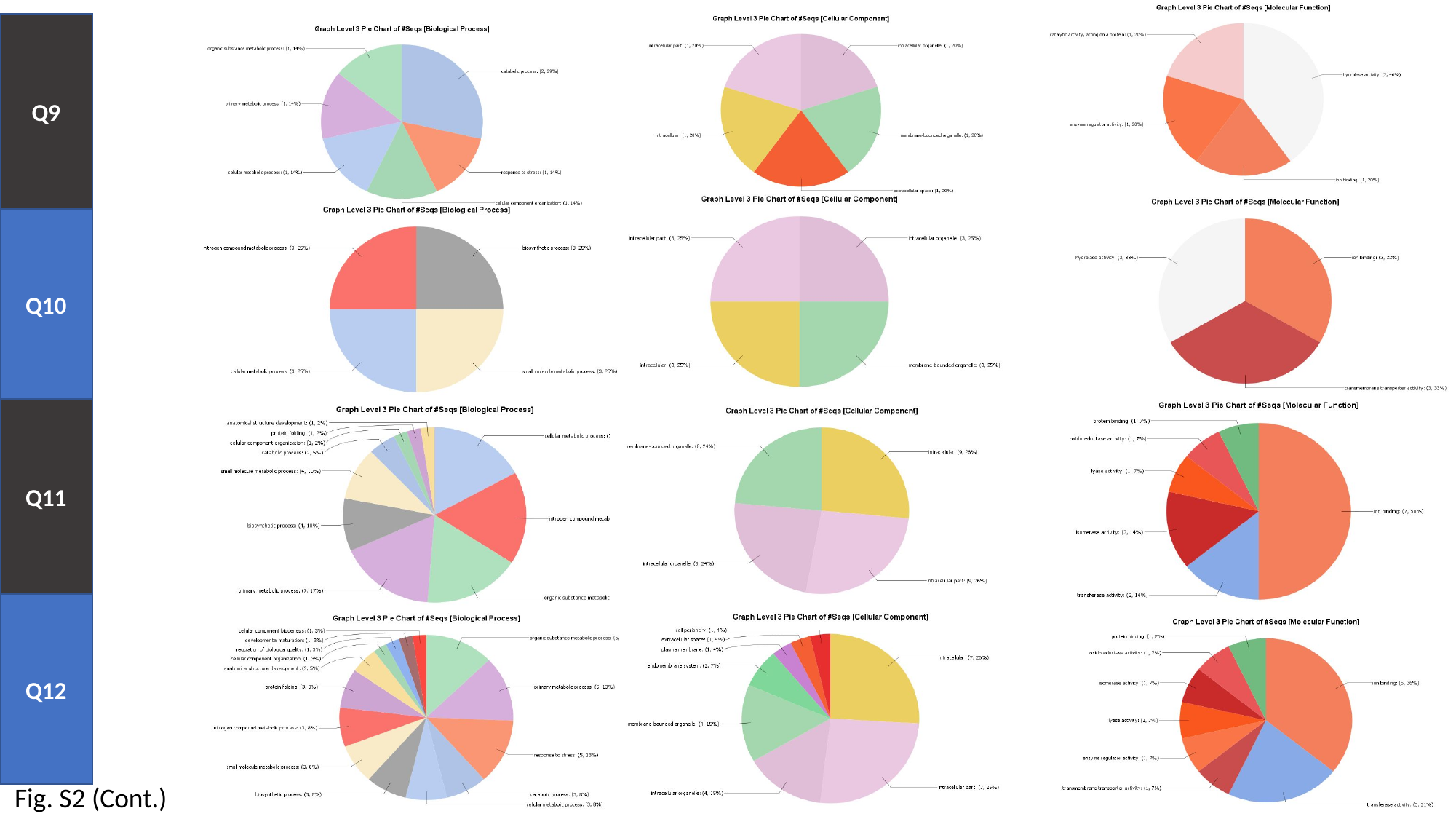

Q9
Q10
Q11
Q12
Fig. S2 (Cont.)

## Slide 5
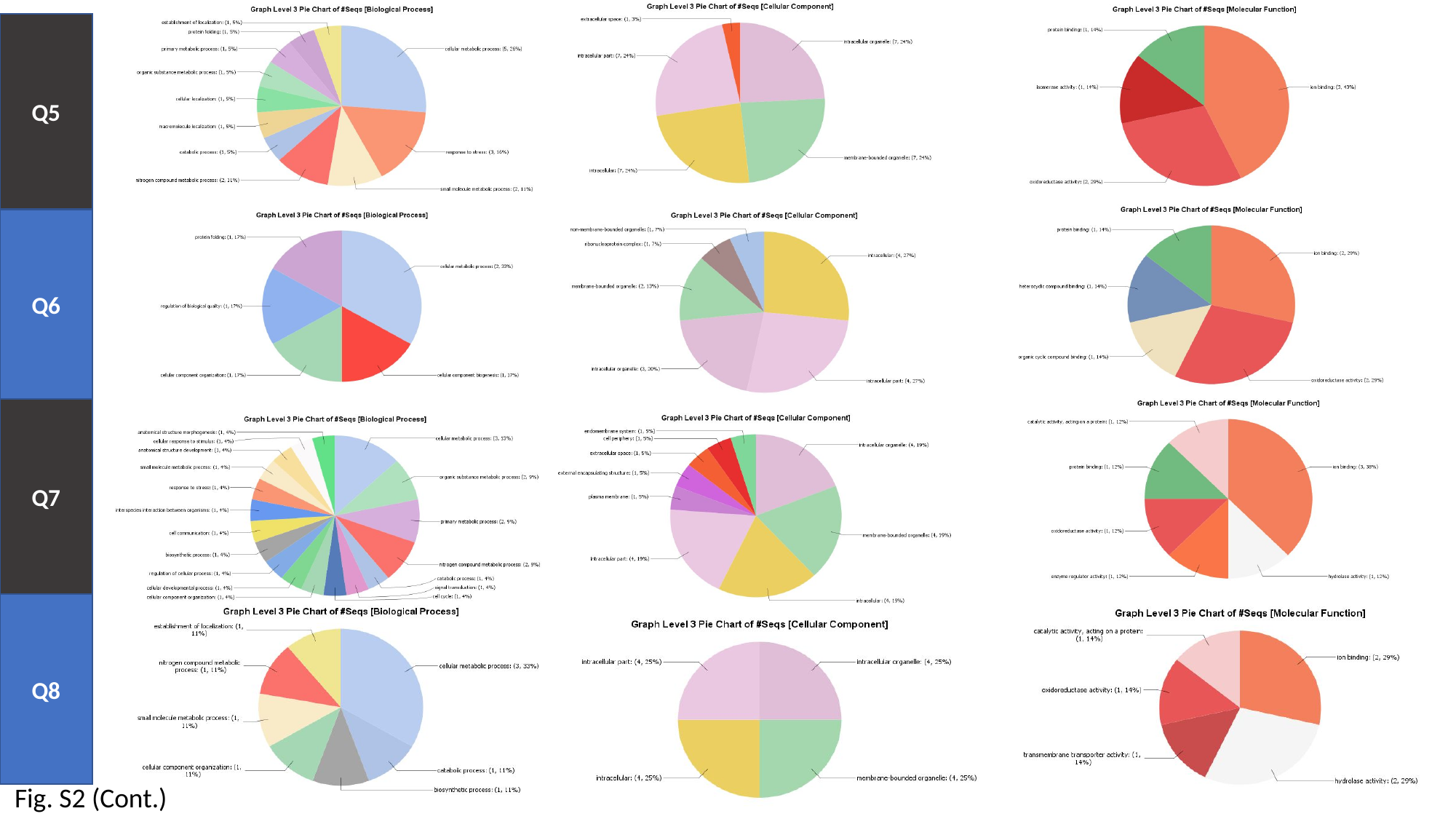

Q5
Q6
Q7
Q8
Fig. S2 (Cont.)
